# Expression of ERCC1, TYMS, TUBB3, RRM1 and TOP2A in patients with esophageal squamous cell carcinoma: A hierarchical clustering analysis

**DOI:** 10.3892/etm.2014.1659

**Published:** 2014-04-02

**Authors:** YONGKANG YU, SHENG DING, YU LIANG, YIFENG ZHENG, WEI LI, LIE YANG, XIUSHAN ZHENG, JIANQING JIANG

**Affiliations:** Department of Thoracic Surgery, General Hospital of Chengdu Military Region of People’s Liberation Army, Chengdu, Sichuan 610083, P.R. China

**Keywords:** gene expression, hierarchy cluster analysis, esophageal squamous cell carcinoma

## Abstract

The aim of the present study was to investigate the correlation between the expression levels of excision repair cross complementing 1 (ERCC1), thymidylate synthase (TYMS), class III β-tubulin (TUBB3), ribonucleoside-diphosphate reductase (RRM1) and topoisomerase IIα (TOP2A) with the clinical characteristics of patients with esophageal squamous cell carcinoma (ESCC). A total of 29 ESCC tissue samples were collected from patients that had not previously received systematic treatment. The expression levels of ERCC1, TYMS, TUBB3, RRM1 and TOP2A were determined using a microarray technique, while Spearman’s rank correlation analysis was used to determine the strength of the correlations between the expression levels of the biomarkers and the pathogenesis of esophageal cancer. High expression levels of TYMS and TOP2A were observed in 24% of the samples and high expression levels of TUBB3 and RRM1 were identified in 7% of the samples. Hierarchical clustering analysis of these biomarkers enabled the samples to be grouped. Group 1 patients exhibited low expression levels of TYMS, RRM1 and TOP2A and high expression of ERCC1 and TUBB3, while group 2 samples had low expression levels of ERCC1 and TUBB3 and high expression levels of TYMS, RRM1 and TOP2A. Analysis using Fisher’s exact test demonstrated a statistically significant difference in the severity of carcinoma invasion between the two groups (P<0.05), however, no significant differences were identified with regard to the clinical stage or lymphatic metastasis (P>0.05). Therefore, hierarchical clustering analysis indicated that the expression levels of ERCC1, TYMS, TUBB3, RRM1 and TOP2A were closely associated with the clinical characteristics of patients with ESCC.

## Introduction

Esophageal cancer (EC) is the third most common type of gastrointestinal malignancy and has been considered as a leading cause for cancer-induced mortality worldwide. As with other tumors, the outcome for patients with EC has been hypothesized to be strongly associated with the stage at initial diagnosis (1). A five-year survival rate of 57–78% has been reported in patients with early-stage EC, however, for patients with locally advanced EC, a lower five-year survival rate of <15% has been reported, despite attempts to treat the cancer by resection (2).

Recent studies have been conducted with regard to the screening of tumor biomarkers in the pathogenesis and prognosis of various types of carcinomas. For instance, ribonucleoside-diphosphate reductase (RRM1), which encodes the regulatory subunit of ribonucleotide reductase, is hypothesized to be involved in the pathogenesis and development of carcinomas. Furthermore, low expression of RRM1 is associated with a poor survival rate among patients with non-small-cell lung cancer (NSCLC) (3). The excision repair cross complementing 1 (ERCC1) gene encodes the nucleotide excision repair protein, which is involved in the repair of radiation- and chemotherapy-induced DNA damage. According to previous studies, ERCC1 mRNA expression levels are associated with non-response and/or survival rates of patients with colon cancer and NSCLC (4,5). TYMS, the gene that encodes thymidylate synthase, has been demonstrated to be an independent prognostic biomarker of patients that have undergone 5-fluorouracil chemotherapy for the treatment of gastrointestinal tumors (6). Class III β-tubulin (TUBB3) encodes a neuron-specific protein and is considered to be a marker of cancer severity. In addition, high expression levels of TUBB3 have been shown to correlate with low response rates in patients with NSCLC (7). The topoisomerase IIα (TOP2A) gene encodes an enzyme that is involved in DNA replication, and is associated with the sensitivity to anthracycline therapy in various carcinomas. In a number of studies, associations between a combination of two or more biomarkers and the pathogenesis of carcinomas have been investigated (3,8). However, to the best of our knowledge no study has been conducted using ERCC1, TYMS, TUBB3, RRM1 and TOP2A simultaneously in esophageal squamous cell carcinoma (ESCC).

Therefore, in the present study, the expression levels of ERCC1, TYMS, TUBB3, RRM1 and TOP2A were analyzed in patients with ESCC, with the aim of investigating the correlation between these five biomarkers and the pathogenesis and development of ESCC.

## Patients and methods

### Patients

A total of 29 male ESCC patients admitted to the Department of Thoracic Surgery at the General Hospital of Chengdu Military Region of People’s Liberation Army (Chengdu, China) between December 2011 and December 2012 were included in the study. The patients were aged between 40.5 and 69.3 years (median age, 54.9 years). Only three patients (10.34%) reported a history of smoking. Patients with ESCC who were suitable for surgical resection and had not received systematic treatment prior to resection were included in the study. Patients were also required to volunteer to participate in the gene test. All the patients provided written informed consent and the study was approved by the Ethics Committee of the General Hospital of Chengdu Military Region of People’s Liberation Army.

### Sample collection

Prior to the sample collection, a thoracotomy was conducted under general anesthetic using cisatracurium besilate (Tianjin Elong Co., Ltd, Tianjin, China), fentanyl injection (Jiangsu Nhwa pharmaceutical corporation, Xuzhou, China) or midazolam hydrochloride (3B Scientific Corporation, Wuhan, China). In total, 29 samples were obtained from the central region of each tumor mass. The samples were fixed for 16–24 h using 10% formalin and were labelled according to the ID numbers of the patients, which were designated as 1–29.

### MicroRNA expression profiling analysis

Total RNA was extracted using an RNA isolation kit (Qiagen, Inc., Valencia, CA, US), according to the manufacturer’s instructions. The RNA integrity number was calculated to analyze RNA integration using an Agilent Bioanalyzer 2100 (Agilent Technologies, Santa Clara, CA, USA). All the microRNA microarray experiments were conducted using an Agilent Human miRNA microarray kit (version 16.0; Agilent Technologies). Subsequently, 100 ng total RNA was hybridized for each sample and processed according to the manufacturer’s instructions. The microRNA arrays were scanned using a G2565BA scanner (Agilent Technologies) and the images were analyzed using Agilent Feature Extraction software (version 10.7). Raw data were normalized using the Quantile algorithm function of the Gene Spring Software 11.0 (Agilent Technologies).

### Statistical analysis

SAS 9.2 software (SAS Institute, Inc., Cary, NC, USA) was used to perform data analysis. Spearman’s rank correlation analysis was used to determine the strength of the associations between the expression levels of the biomarkers and the pathogenesis of ESCC. Fisher’s exact test was conducted to determine the contingency of the data, where P<0.05 was considered to indicate a statistically significant difference.

## Results

### Patient characteristics

Invasion evaluation was performed, which revealed that ~76% of the patients were diagnosed with a level two or three depth of invasion (Table I). In addition, levels one and two lymph-node metastasis were identified in 14 patients (48.28%). According to the clinical stages classified by the Union for International Cancer Control (UICC), the numbers of patients in a UICC stage II (A/B) or III (A/B) were 21 (72.41%) and 8 (27.59%), respectively.

### Expression of the biomarkers

High expression levels of TYMS and TOP2A were observed in 24.14% of the samples, while high expression levels of TUBB3 and RRM1 were observed in 6.9% of the samples (Table II). Only low and moderate expression levels of ERCC1 were observed in the samples; high expression was not identified.

### Correlation analysis

Spearman’s rank correlation analysis was performed to investigate the correlation between the expression levels of the biomarkers and the clinical features of the patients. The results indicated that the expression of ERCC1 positively correlated with the severity of tumor invasion (r=0.47, P=0.0102). In addition, the expression level of ERCC1 was negatively associated with lymphatic metastasis (r=−0.39, P=0.0357). The expression level of TYMS exhibited a negative correlation with tumor invasion (r=−0.47, P=0.0098) and the clinical stage of carcinoma (r=−0.43, P=0.0191). With regard to TUBB3 expression levels, a negative association was observed with lymphatic metastasis (r=−0.42, P=0.0231). No significant correlation was identified between the expression levels of TOP2A and RRM1 with the clinical features of the patients (Table III).

### Cluster analysis of gene expression levels

**Figure 1:**
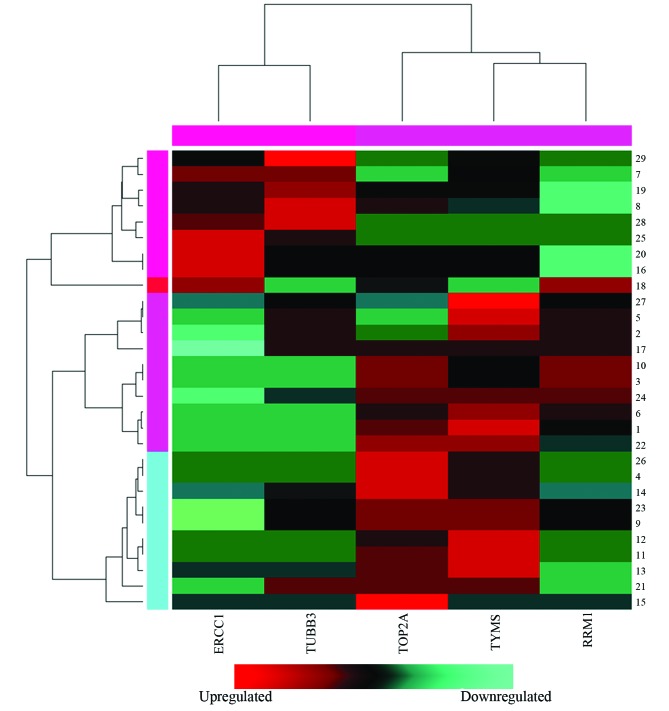
MicroRNA expression patterns in the ESCC patients. 1–29 are the sample IDs. ESCC, esophageal squamous cell carcinoma.

To investigate the association between the gene expression levels of the biomarkers and the clinical features of the patients, cluster analysis was performed on ERCC1, TYMS, TUBB3, RRM1 and TOP2A. High expression levels of ERCC1 and TUBB3 were identified in the samples with an ID of 6, 7, 15, 18–20, 25, 28 and 29, while low expression levels of ERCC1 and TUBB3 were observed in these samples (Fig. 1). With regard to the other samples, comparatively high expression levels of TYMS, RRM1 and TOP2A were demonstrated, while low expression levels of ERCC1 and TUBB3 were identified. Based on these results, the samples were divided into two groups. Group 1 patients exhibited low expression levels of TYMS, RRM1 and TOP2A and high expression levels of ERCC1 and TUBB3, while group 2 exhibited low expression levels of ERCC1 and TUBB3 and high expression levels of TYMS, RRM1 and TOP2A. Subsequently, Fisher’s exact test was conducted to analyze the expression levels of the biomarkers in these groups, which revealed a statistically significant difference in the severity of carcinoma invasion between the two groups (P<0.05, Table IV). However, no significant differences were identified between groups 1 and 2 with regard to clinical stage or lymphatic metastasis (P>0.05).

## Discussion

ESCC, a disease prevalent in China, has been reported to be a consequence of polymorphisms of multiple interacting genes and gene-environment interactions. However, to the best of our knowledge, no study has been conducted investigating the expression of ERCC1, TYMS, TUBB3, RRM1 and TOP2A in patients with ESCC. In the present study, the expression levels of ERCC1, TYMS, TUBB3, RRM1 and TOP2A were evaluated simultaneously. The results indicated that the expression levels of ERCC1, TYMS, TUBB3, RRM1 and TOP2A were closely associated with the clinical characteristics observed in patients with ESCC.

Increasing evidence has demonstrated that the expression levels of ERCC1, TYMS, TUBB3, RRM1 and TOP2A are closely correlated with the pathogenesis of carcinomas. For example, the aberrant regulation of ERCC1, TYMS, TUBB3, RRM1 and TOP2A is associated with the abnormal proliferation of cancer cells, according to the hallmarks of cancer that were proposed previously (9). Furthermore, Zhang *et al* (10) identified that the polymorphism and aberrant expression of TYMS may be associated with a susceptibility to ESCC and gastric cardiac adenocarcinoma. In addition, increased expression of ERCC1 has been hypothesized to be closely associated with a reduced survival rate in patients with ESCC. In a review that evaluated the biomarkers for predicting the response and/or prognosis of ESCC patients treated with neoadjuvant chemoradiation therapy, expression levels of ERCC1 were considered to be an independent risk factor for poor outcomes (11). With regard to the expression levels of TUBB3 in carcinomas, Levallet *et al* (12) demonstrated that TUBB3 expression was associated with nonsquamous cell carcinoma. A previous study investigated the association between RRM1 expression levels and sensitivity to gemcitabine in ESCC cell lines, and a close correlation was identified (13). Hanagiri *et al* (14) observed high expression of TOP2A in 55.2% of the tumor specimens that were analyzed. However, the expression of TOP2A exhibited no correlation with the clinical features of the specimens, including the differentiation, depth of tumor invasion and lymph node metastasis.

Single gene analysis indicated that ERCC1 and TYMS expression levels were associated with the depth of tumor invasion, while TUBB3 expression was associated with the lymphatic metastasis of ESCC. However, single gene analysis has certain disadvantages in predicting the association between biomarker expression levels and the pathogenesis of ESCC, specifically with regard to neglecting gene interactions. Therefore, the association between ERCC1, TYMS, RRM1, TUBB3 and TOP2A expression levels with the pathogenesis and development of ESCC was investigated using a clustered analysis in terms of tumor invasion and lymphatic and distal metastasis. From the results, it was possible to divide the samples into two groups. Group 1 exhibited low expression levels of TYMS, RRM1 and TOP2A and high expression levels of ERCC1 and TUBB3. By contrast, group 2 specimens had low expression levels of ERCC1 and TUBB3 and high expression levels of TYMS, RRM1 and TOP2A. Fisher’s exact test revealed a statistically significant difference in the severity of carcinoma invasion between the two groups (P<0.05). However, no statistically significant differences were observed with regard to the clinical stages and lymphatic metastasis (P>0.05).

In conclusion, hierarchical clustering analysis of the biomarkers revealed low expression levels of TYMS, RRM1 and TOP2A and high expression levels of ERCC1 and TUBB3 in certain individuals. By contrast, low expression levels of ERCC1 and TUBB3 and high expression levels of TYMS, RRM1 and TOP2A were observed in other patients. Therefore, these observations indicated that the expression levels of ERCC1, TYMS, TUBB3, RRM1 and TOP2A were closely associated with the clinical characteristics of patients with ESCC.

## Figures and Tables

**Table I:** Clinical features of the patients.

Clinical feature	Cases, n (%)
Depth of invasion	
Level 1	5 (17.24)
Level 2	10 (34.48)
Level 3	12 (41.38)
Level 4	2 (6.90)
Lymphatic metastasis	
Level 0	15 (51.72)
Level 1	11 (37.93)
Level 2	3 (10.34)
Clinical stage^a^	
IIA	5 (17.24)
IIB	16 (55.17)
IIIA	7 (24.14)
IIIB	1 (3.45)

a According to the Union for International Cancer Control.

**Table II:** Gene expression levels interpreted from the microarray analysis.

Gene	Low	Low to moderate	Moderate	Moderate to high	High
ERCC1, n (%)	14 (48.28)	5 (17.24)	8 (27.59)	2 (6.90)	0 (0.00)
TYMS, n (%)	4 (13.79)	8 (27.59)	6 (20.69)	4 (13.79)	7 (24.14)
TUBB3, n (%)	10 (34.48)	8 (27.59)	3 (10.34)	6 (20.69)	2 (6.90)
RRM1, n (%)	16 (55.17)	3 (10.34)	4 (13.79)	4 (13.79)	2 (6.90)
TOP2A, n (%)	6 (20.69)	7 (24.14)	7 (24.14)	2 (6.90)	7 (24.14)

ERCC1, excision repair cross complementing 1; TYMS, thymidylate synthase; TUBB3, class III β-tubulin; RRM1, ribonucleoside-diphosphate reductase; TOP2A, DNA topoisomerase IIα.

**Table III:** Spearman’s rank correlation analysis of gene expression levels and clinical features.

Clinical feature	ERCC1	TYMS	TUBB3	TOP2A	RRM1
Depth of tumor invasion	0.4692	−0.4718	0.2293	−0.2498	−0.0776
P-value	0.0102	0.0098	0.2315	0.1911	0.6888
Lymphatic metastasis	−0.392	0.0127	−0.4205	0.0039	−0.1716
P-value	0.0357	0.9481	0.0231	0.9838	0.3733
Clinical stage	0.0376	−0.4305	−0.1729	−0.2975	−0.2832
P-value	0.8463	0.0197	0.3696	0.1170	0.1365

ERCC1, excision repair cross complementing 1; TYMS, thymidylate synthase; TUBB3, class III β-tubulin; RRM1, ribonucleoside-diphosphate reductase; TOP2A, DNA topoisomerase IIα. P<0.05 was considered to indicate a statistically significant difference.

**Table IV:** Analysis between biomarker expression levels and clinical features using Fisher’s exact test.

Clinical feature	Group 1, n (%)	Group 2, n (%)	P-value
Depth of invasion			0.02
Level 1	0 (0)	5 (25)	
Level 2	1 (11.11)	9 (45)	
Level 3	7 (77.78)	5 (25)	
Level 4	1 (11.11)	1 (5)	
Lymphatic metastasis			1
Level 0	5 (55.56)	10 (50)	
Level 1	3 (33.33)	8 (40)	
Level 2	1 (11.11)	2 (10)	
Clinical stage^a^			0.19
IIA	0 (0)	5 (25)	
IIB	5 (55.56)	11 (55)	
IIIA	3 (33.33)	4 (20)	
IIIB	1 (11.11)	0 (0)	

a According to the Union for International Cancer Control.
